# Healthcare-associated transmission of Panton-Valentine leucocidin positive methicillin-resistant *Staphylococcus aureus*: the value of screening asymptomatic healthcare workers

**DOI:** 10.1186/s12879-018-3404-2

**Published:** 2018-09-27

**Authors:** Panagiotis Papastergiou, Eleni Tsiouli

**Affiliations:** 1 Microbiology Department, NRP Innovation Centre, Norwich Research Park, Norfolk and Norwich University Hospital, Colney, Norwich, NR4 7GJ UK; 2 Infection Prevention Control/ Microbiology Department, The Queen Elizabeth Hospital King’s Lynn, Gayton Road, King’s Lynn, PE30 4ET UK

**Keywords:** Healthcare worker, Methicillin-resistant *Staphylococcus aureus*, MRSA, nosocomial, Infection control, Occupational health, Panton–Valentine leucocidin, PVL

## Abstract

**Background:**

Three patients hospitalised in the coronary care unit of a general district hospital (England, UK) were tested positive for Panton-Valentine leucocidin methicillin-resistant *Staphylococcus aureus* colonisation during their routine weekly screening for methicillin-resistant *Staphylococcus aureus* (MRSA). The isolates were indistinguishable and all three patients have previously had negative screening tests. The outbreak investigation team considered exploring the possibility of PVL-MRSA transmission from members of staff to the patients and potentially between members of staff.

**Method:**

As part of the investigations, healthcare workers on coronary care unit and intensive care unit were screened for MRSA carriage.

**Results:**

Among 134 screened healthcare workers, five staff members (3.7%) were MRSA colonised. Among these isolates, four were Panton-Valentine leukocidin positive. However, only two healthcare workers had an indistinguishable isolate with the isolate identified among the colonised patients. Decolonisation treatment was offered to all colonised patients and healthcare workers.

**Conclusion:**

In low MRSA prevalence settings, healthcare workers may be a reservoir of MRSA and an important potential source of transmission to patients. Screening and decolonisation of colonised healthcare workers may provide a valuable strategy in managing linked hospital acquisitions and reduce the risk of occupationally acquired complications. MRSA mass screen of healthcare workers should be considered in transmission with a strain that has a potentially increased virulence, such as Panton-Valentine leucocidin methicillin-resistant *Staphylococcus aureus*.

## Background

Preventing transmission of methicillin-resistant *Staphylococcus aureus* (MRSA) is a high priority for infection control and prevention teams. An outbreak of MRSA in healthcare settings can be linked to colonised healthcare workers [1–6].

Panton-Valentine leukocidin (PVL) is an exotoxin transmitted by bacteriophages carried by some MRSA and methicillin sensitive *Staphylococcus aureus* (MSSA) strains. PVL exotoxin has been associated epidemiologically with virulent, transmissible strains of *S. aureus*, including community-associated (CA) MRSA [7, 8]. MRSA and PVL-S.aureus infections can cause occupational health problems in healthcare workers (HCWs), including long-term morbidity and mortality [9, 10].

All inpatients in our hospital undergo an admission MRSA screen and subsequent once a week MRSA screen which includes the nose, axillae, and any skin lesions.

In September 2016, all patients in our three bedded coronary care unit (CCU) were tested positive during their weekly routine MRSA screen (nose, axilla, groin) three days after their admission to the unit when they were tested negative. This fact indicated that the transmission was more likely to have occurred within this period of three days. The isolates had identical phenotypic susceptibility and were resistant only to beta-lactam antibiotics. All identified MRSA isolates were sent to the Public Health England reference laboratory (Colindale London, UK) for typing. The results showed that all three isolates had indistinguishable staphylococcal protein A (spa) types and spa repeat succession patterns and in addition, they were PVL (Luk-PV) positive. The three bedded CCU is adjoining to the thirteen bedded intensive care unit (ICU) with no door or walls between them and the two units are sharing members of staff. As part of the investigations we identified one more patient in ICU colonised with MRSA from a wound swab two weeks prior to the incident. The ICU patient was discharged one week prior the CCU transmission. This MRSAisolate was proven to be indistinguishable from the one identified among the colonised CCU patients (phenotypical susceptibility, spa type and spa repeat succession patterns).

Mass screening of asymptomatic HCWs for MRSA colonisation is routinely not recommended in the UK. Screening of HCWs is indicated if transmission continues or if the epidemiological aspects of an outbreak are unusual, or if they suggest persistent MRSA carriage by staff [11]. In addition, screening of staff should be performed if a case of PVL-SA infection was acquired in hospital [8].

The outbreak investigation team considered exploring the possibility of PVL-MRSA transmission from members of staff to the patients and potentially between members of staff. Therefore, we contacted an investigation including prospective MRSA testing between HCWs and we present the outcomes of this study.

## Methods

### Enrolment

A letter was sent to all involved healthcare staff including housekeepers, domestics regular, agency and visiting HCWs from other specialities who have been involved with those patients’ care during the three days period between admission to the CCU and MRSA detection. Those were identified by the duty roster and the patient’s notes. HCWs were asked to be screened for MRSA carriage in a confidential manner. All staff members on CCU and ICU, including agency staff, housekeepers and domestics were screened for MRSA. In addition, visiting clinicians during the three days period (between admission screen and MRSA positivity) were included in the screening. Samples from nose, axilla, throat and skin rash or wounds (if applicable) were acquired from all HCWs and were sent to the laboratory under a code to ensure pseudonymisation.

### Laboratory testing

Screening swabs were inoculated into Contrast™ MRSA broths (Oxoid, UK). Multiple swabs (maximum 4) from the same patient or staff were pooled into a single broth. The samples were processed in the microbiology laboratory of Norfolk and Norwich University Hospital. *Staphylococcus aureus* ATCC33591 strain was used as a control to monitor the test performance. After incubation at 35–37 °C for 16–24 h, suspected positive broths and control broths (ATCC33591) were subcultured onto chromogenic Brilliance MRSA-2 agar (Oxoid, UK). Plates were incubated at 37 °C for 18–20 h. Suspected denim blue colonies were picked onto blood agar for purity. Antibiotic sensitivity testing was performed by EUCAST disk diffusion test methodology. Identification of MRSA isolates was confirmed by VITEK MS (bioMérieux) and antibiotic sensitivities were confirmed by VITEK 2 XL (bioMérieux). The MRSA testing method was applied to both patients and HCWs tested.

All MRSA strains identified at the laboratory were sent for typing to the Antimicrobial Resistance and Healthcare Associated Infections Reference Unit (AMRHAI) in PHE Colindale (London, UK).

### Follow up

The MRSA colonised HCWs were referred to the occupational health department. Expert consultation and decolonisation with octenidine dihydrochloride body wash, mupirocin nasal ointment and mouthwash with 0.2% chlorhexidine gluconate were offered to the identified MRSA colonised HCWs. The decolonisation was considered successful if the staff member had three consecutive, one week apart, negative MRSA screens. Decolonisation of close household member’s and the follow-up screens for the discharged patient was overseen by primary care and the PHE authorities.

## Results

Among 134 screened HCWs, five (3.7%) were colonised with MRSA. The spa typing data showed that the isolates from the 5 HCWs displayed three different spa types and spa repeat succession patterns. Two HCWs were colonised with the epidemic MRSA-PVL isolate that displayed the indistinguishable spa type t019 (spa repeat succession pattern 08–16–02-16-02-25-17-24), identified among all four patients (three CCU patient and one recently discharged ICU patient). Further two HCWs were colonised with a MRSA-PLV isolate that displayed the spa type t002 (spa succession pattern 26–23–17-34-17-20-17-12-17-16). The 5th HCW was colonised with a MRSA isolate that was not PLV positive that displayed the spa type t843 (spa repeat succession pattern 04–82–17-25-17-25-25-16-17). The number of screened HCWs, CCU, ICU patients and positive PVL-MRSA cases are summarised in Table 1.

**Table 1:** Number of screened HCWs, CCU, ICU patients and positive PVL-MRSA cases

	CCU Patients	ICU Patients	HCWs	Site of screen/ colonisation
Patient or HCWs screened	3	13	134	Nose, axilla, groin, wound site (if applicable)
PVL-positive MRSA spa type t019 cases	3	1	2	Nose, axilla, groin, wound site
PVL-positive MRSA spa type t002 cases	0	0	2	Nose, axilla, groin
MRSA (PVL negative) spa type t843 cases	0	0	1	Nose, axilla, groin

## Discussion

Two HCWs were colonised with the MRSA PVL-S.aureus strain spa type t019 identified among the colonised patients, supporting the hypothesis of transmission between patients and healthcare staff.

The prevalence of MRSA colonisation among tested HCW in the CCU/ICU was 3.7%. The published MRSA colonisation rates among healthcare staff vary significantly with some countries reporting a relatively high MRSA carriage rate and PVL gene-containing cases among their healthcare staff [12, 13]. The UK studies conducted in endemic hospital settings report non-outbreak carriage rates between 0 and 15% [14]. Surveys of healthcare staff at medical institutions in Germany estimate the MRSA prevalence between 0.4 to 4.5% [15]. Other researchers estimated the prevalence of MRSA in HCWs around 4.6% and their estimated MRSA prevalence among HCWs in non-outbreak settings was no higher than carriage rates estimated for outbreak [16].

Since all in-patients from high-risk units at the hospital undergo a once weekly routine screen for MRSA, in addition to their admission screen, we were able to determine that the MRSA transmission to these three patients occurred within a relatively short period of 72 h. The fact that two HCWs were tested positive with an indistinguishable isolate suggests that they were part of the transmission. Two weeks before the incidence, a patient repatriated to ICU from another healthcare facility was found to be MRSA colonised. The MRSA was isolated only from the wound site and was not detected from any other site (nose, axilla, groin) during the routine screen and follow up screens. This isolate was proven to be indistinguishable from the one identified among the CCU colonised patients (phenotypical susceptibility, spa type and spa repeat succession patterns). Because the patient’s wound site was not initially swabbed during the re-admission, it is not clear if the patients wound was already colonised or became colonised during the re-admission. The ICU patient was discharged a few days before the CCU transmission and no further MRSA cases were detected during the MRSA screens among the ICU patients. The HCWs colonised with the epidemic strain had contact with each other and one HCW had professional contact with the colonised ICU patient and the three CCU patients. Nonetheless, we were unable to identify whether the patient in ICU, two weeks before the incidence, or one of the two HCWs was the “case zero” in this transmission.

Screening of HCWs is recommended if a case of PVL-*S.aureus* infection was acquired in hospital [8] or if transmission continues or if the epidemiological aspects of an outbreak are unusual, or if they suggest persistent MRSA carriage by staff [11]. In total six patients and staff were colonised with the same indistinguishable PVL-*S.aureus* isolate, however none among patients or staff had an infection. Exposure to an untreated MRSA carrier is associated with an increase in MRSA acquisition risk and decolonisation can reduce transmission [17].

Occupationally acquired colonisation with methicillin-resistant *Staphylococcus aureus* (MRSA) is an issue of increasing concern that should be addressed. Eradication of colonisation/carriage of healthcare workers should be attempted [11]. In addition, MRSA infection can cause occupational health problems in HCWs that may lead to long-term health problems [10] and occasionally may lead to a fatal outcome [9]. The spread of MRSA isolates with additional potent virulent factors such as PVL require further attention. There is a duty to protect the health, safety and welfare of hospital employees. Irrespectively, if HCWs are potentially the source of an outbreak of MRSA or are colonised with a *S.aureus* strain that has increased virulence, such as PVL positive, then decolonisation should be considered.

The identified advantages and challenges, during our mass-staff screen, are summarised in Table 2. The information was obtained during compliance observations in ICU/CCU, verbal feedback and discussions with occupational health staff, involved staff representatives and first-hand discussion with affected HCWs.

**Table 2:** Identified advantages and challenges, during our mass staff screen

Advantages	Challenges
• Epidemiology outbreak investigation - determining the most likely source. • Protect the health, safety and welfare of patients, HCWs and their families. • Raises awareness among HCWs. • Improves knowledge. • Improves infection control compliance among HCWs.	• Testing cost. • Time and operational cost for related departments. • Fear of stigmatisation if confidentiality and anonymity protection fails. • Psychological pressure and qualms of conscience among HCWs. • Easeless, anxiety for family safety.

The letter explaining the concerns and the rationale for the screen was sent to all involved healthcare staff. This provided clarity and contributed significantly to the HCWs engagement in the mass screen process. All identified staff members participated.

The identification of two different MRSA-PVL spa types/spa repeat succession patterns (t019, 08–16–02-16-02-25-17-24 and t002, 26–23–17-34-17-20-17-12-17-16) among the staff was an unexpected finding. Two HCWs were linked with the identified t019 spa type PVL-MRSA transmission. The spa type t019 has been associated with PLV-MRSA belonging to MLST clonal complex 30 reported in the South-West Pacific region and seen previously in the UK [18]. Two further HCWs were asymptomatic carriers of a t002 spa type MRSA that was PVL positive. However, we didn’t identify any transmission of that spa type (t002) among patients. There is a possibility that their colonisation was recently acquired abroad. It is also possible that the high hand hygiene and environmental cleaning standards in CCU/ICU, likely reduced further spread.

We considered that potential risk factors for this MRSA transmission was possible fatigue (long shifts) and a mild upper respiratory infection with nasal discharge among one of the MRSA colonised HCW. The risks posed by high workloads can be mitigated by good compliance with infection control measures, training and adequate staffing ratios in the ICU [19]. In addition, it’s likely that having a concurrent respiratory infection leads one to be more likely to spread MRSA.

It is worth mentioning that until to date, approximate 21 months later, no further cases of PVL-MRSA have been identified at the CCU and ICU.

Our investigation has limitations. Environmental samples were not obtained since the PVL result was available several days after the transmission occurred and the decontamination of the environment and equipment was completed at CCU and ICU. In addition, we acknowledge that spa typing in MRSA outbreak investigations may be limited due to its low discriminatory power in comparison to whole genome sequencing.

## Conclusion

In conclusion, in low MRSA prevalence settings healthcare workers may be a substantial reservoir of MRSA and an important potential source of transmission to patients. Screening HCWs and decolonisation of colonised members may provide a valuable strategy in managing linked hospital acquisitions and reduce the risk for occupationally acquired complications. MRSA screen and decolonisation should be considered if a healthcare worker is identified as a potential source of a transmission of MRSA. In addition, if healthcare workers are found to be colonised with a *S.aureus* stain that has a potentially increased virulence, such as PVL toxin, then decolonisation should be offered.

## Abbreviations

CCU: Coronary care unit
HCW: Healthcare workers
ICU: Intensive care unit
MRSA: Methicillin-resistant *Staphylococcus aureus*
MSSA: Methicillin-sensitive *Staphylococcus aureus*
PHE: Public Health England
PVL: Panton-Valentine leukocidin
